# The Role of the Cerebellum in Skin-Picking Disorder

**DOI:** 10.1007/s12311-018-0957-y

**Published:** 2018-06-22

**Authors:** Albert Wabnegger, Anne Schienle

**Affiliations:** Clinical Psychology, University of Graz, BioTechMed, Universitätsplatz 2/III, A-8010 Graz, Austria

**Keywords:** Functional MRI, Structural MRI, gPPI, Skin-picking disorder

## Abstract

Previous research indicated that the cerebellum is involved in psychopathologies with body-focused repetitive behaviors. The present study investigated whether patients with a diagnosis of skin-picking disorder (SPD) also show altered cerebellar structure and function. Structural as well as functional MRI data from 30 SPD patients and 31 controls were analyzed. The fMRI approach compared cerebellar activity and connectivity between the two groups during scratching and caressing of a small skin area on the arm. Relative to controls, SPD patients were characterized by reduced gray matter volumes in the left cerebellar lobules V and VI. During picking (relative to caressing), SPD patients displayed increased activation of the left crus I, which showed enhanced coupling with the left ventrolateral prefrontal cortex (VLPFC). This study provides the first evidence that SPD patients display structural as well as functional abnormalities in specific subregions of the cerebellum related to motor (V) and affective-cognitive functions (VI, crus I). The SPD-related altered cerebellar connectivity with an area implicated in affect control (VLPFC) fits nicely to the model of pathological skin picking as a maladaptive emotion regulation strategy.

## Introduction

The core symptom of skin-picking disorder (SPD) is a specific type of body-focused repetitive behavior causing physical injury [1]. SPD patients display ongoing and excessive picking of their skin that is triggered by seeing or touching dermatological irregularities (e.g., scabs, bug bites, pimples, ingrown hairs). This type of skin manipulation is labeled “focused skin-picking” [2]. It is preceded by feelings of tension and urge to pick, while the removal of skin irregularities is usually accompanied by tension reduction, relief, or gratification [3].

Some SPD patients do not only engage in focused skin picking but additionally show automatic picking [1]. They report that they unconsciously manipulate their skin, and only much later notice that they have been picking (e.g., because of pain or bleeding). For both types of skin picking, the consequences can be serious. The picking creates or worsens skins lesions. Some patients are covered with sores and scars and experience complications, such as infections. Besides physical injury and associated disfigurement, SPD causes clinically significant distress and impairment in important areas of functioning [4].

The two mentioned types of skin manipulation (focused and automatic) are characterized by relatively stereotyped, coordinated, and repetitive motor movements. The picking is typically executed with the fingernails (especially with the index finger of the dominant hand), and the most common scratched sites are the face, arms, and hands, although other body parts can also be a target [5]. Thus, pathological skin picking has a strong motor component, which however has hardly been addressed by neurobiological research so far.

In general, limited knowledge exists regarding the underlying neuronal mechanisms of SPD. Previous structural neuroimaging studies have found reduced integrity of white matter tracts connecting anterior cingulate cortices in SPD patients [6], a greater volume of the bilateral nucleus accumbens, and reduced cortical thickness in the right frontal areas, compared to those in control participants and patients with trichotillomania [7]. In contrast, Harries et al. [8] detected no differences in gray matter volume between SPD patients and controls.

Functional neuroimaging investigations have focused on cognitive and emotional processing in SPD [6, 7, 9–11]. These studies have pointed to alterations regarding activation of the basal ganglia, the insula, and the anterior cingulate cortex during executive planning tasks and the viewing of affective pictures.

To the best of our knowledge, there is only one fMRI study with a symptom provocation design [12]. SPD patients and control participants were instructed to either scratch or gently stroke a small skin area on their arms. Female SPD patients showed less activation in the middle frontal gyrus and primary/secondary somatosensory cortices during caressing relative to scratching (and resting) than controls. This symptom provocation study hinted at a reduced sensitivity of pleasant touch in women with SPD. In contrast, activation in the chosen motor regions of interest (basal ganglia, SMA) did not differ between patients and controls.

However, one subcortical region that has classically been viewed as being dedicated to the control of motor behavior was not investigated: the cerebellum. This region is not a uniform entity but rather a heterogeneous structure that can be divided into three portions and ten lobules (anterior lobe, lobules I–V; posterior lobe, lobules VI–IX; flocculonodular lobe, lobule X; see [13]). Recent research has indicated that the cerebellum contributes to cognitive, affective, and social aspects of behavior in addition to motor functions [14, 15]. These different functions can be realized because the cerebellum has pronounced interconnections with various cortical areas. For example, the cerebellum receives input from virtually all motor cortical regions. Moreover, prefrontal cortex-cerebellar circuits exist, which are involved in the modulation of cognitive-affective processes (e.g., [16]).

Due to this multifunctionality, cerebellar dysfunctions and lesions can lead to cognitive and affective symptoms (e.g., [17]). More specifically, cerebellar dysfunctions have been identified in different psychopathologies including those with body-focused repetitive behaviors, such as trichotillomania (e.g., [18]). This disorder is characterized by repetitive and coordinated motor behaviors of touching and stroking the hair, ultimately culminating in hair extraction. A morphometric study found that trichotillomania patients demonstrated reduced cerebellar cortex volumes relative to controls. The volume reduction referred to both the motor areas of the cerebellum as well as to regions with emotional functions [18].

The present study followed up on this finding and investigated whether SPD patients also show reduced cerebellar volume. In addition, functional MRI data of a previous investigation [12] were reanalyzed in order to find out if the cerebellum is recruited during skin picking and if cerebellar activity as well as connectivity differs between SPD patients and controls. The Spatially Unbiased Infratentorial Template (SUIT [19]) was used which allows an accurate assessment of anatomical details of the cerebellum and its subdivisions.

## Methods

### Participants

Thirty individuals with a primary current diagnosis of SPD (19 women, 11 men) and 31 control participants (16 women, 15 men) were recruited via the outpatient clinic of the university and newspaper advertisements. The participants were on average 34.4 years (SD = 14.7) old; mean duration of education was 10.7 years (SD = 1.7). The groups did not differ in both variables (ps > .10).

All participants were assessed by a board-certified clinical psychologist who confirmed the SPD diagnosis. In addition to the clinical interview, the patients answered the Milwaukee Inventory for the Dimensions of Adult Skin-picking (MIDAS [2]) with two subscales: automatic and focused picking. The mean scores (SD) were as follows: *M*_focused_ = 18.73 (SD = 5.87), *M*_automatic_ = 19.40 (SD = 4.92). The patients reported that they picked their skin on average for 2.3 h/day (SD = 1.88).

Exclusion criteria for the clinical sample were diagnoses of psychosis, substance abuse/dependence, and severe depressive symptoms. Diagnosed comorbidity included major depression (mild to moderate symptoms) in two patients, who received antidepressant medication. Any lifetime diagnosis of a mental disorder led to exclusion from the control group.

After a complete description of the study, written informed consent was obtained. The local ethics committee of the university approved this study, which was carried out in accordance with the ethical principles established in the 2008 Declaration of Helsinki.

### Procedure and Design

The investigation included a structural scan and functional magnetic resonance imaging during skin manipulation. Each SPD patient selected a skin region (with a defined size) located on the arm. In the scanner, the following instructions were given: “scratch region” (scratch the region without causing injury), “caress region” (gently stroke the region in a way most pleasurable for you), and “rest” (put the arm beside the body). The experiment had a block design. Each condition lasted 15 s and was repeated four times. The sequence was random. During the experiment, the participants were monitored via a camera system to check if they adhered to the instructions (which was always the case). Each control participant scratched/caressed the same region as a matched patient.

### MRI: Recording and Analysis

The MRI recording was conducted with a 3-T scanner (Skyra, Siemens, Erlangen, Germany) with a 32-channel head-coil. Functional runs were acquired using an echo-planar imaging protocol (number of slices 35, descending, flip angle = 90°, slice thickness 3 mm; matrix 64 × 64; TE = 30 ms; TR = 2290 ms; FoV: 192 mm; in-plane resolution = 3 × 3 × 3 mm, duration 10:01 min). The parameters for the field map were as follows: number of slices 35, interleaved, flip angle = 60°, slice thickness 3 mm; matrix 64 × 64; TE1 = 4.92 ms, TE2 = 7.38 ms; TR = 400 ms; FoV 192 mm; in-plane resolution = 3 × 3 × 3 mm, duration 0:54 min.

Structural images were obtained using a T1-weighted MPRAGE sequence (number of slices 192, interleaved, flip angle = 8°, slice thickness 0.88 mm, matrix 256 × 256, TR = 1680, TE = 1.89 ms, FoV 224, in-plane resolution 0.9 × 0.9 × 0.9 mm, duration 4:29 min). All analyses were conducted with SPM12 (version 6906; Wellcome Department of Cognitive Neurology, London), the SUIT toolbox (version 3.0), and the generalized PsychoPhysiological Interactions toolbox (gPPI [20]).

The analysis of the functional and structural data for the cerebellum followed the recommendations of the SUIT manual (for details regarding the preprocessing steps for the connectivity analyses, see [12]). The preprocessing pipeline comprised realignment and slice-timing as analysis steps. Three volumes from the beginning of the time series were discarded to account for saturation effects. In the first step, motion correction was conducted (registration to the first volume using realignment and unwarping with an additional field map that should correct additionally for possible field inhomogeneity). Afterwards, acquisition timing was taken into account during the slice-timing step using the middle slice as reference scan. Subsequently, motion- and time-corrected images were used in the first-level analyses. Here, we entered the contrasts “pick,” “caress,” and “rest” into the design matrix to model block-related responses by the canonical hemodynamic response function. Additionally, *t* contrasts were built: “pick–caress,” “pick–rest,” “caress–rest.” Data were high-pass filtered (128 s). An AR(1) process was applied to account for biorhythms and unmodeled neural activity. A Pythagorean transformation of the six motion parameters, which allows the calculation of the magnitude of head movements in two parameters, accounted for motion-induced variance [21]. This resulted in four nuisance regressors (translation-displacement; rotation-displacement; translation-motion; rotation-motion) that were then used as regressors of no interest in further analyses.

After the aforementioned analysis steps, the cerebellum and brainstem were isolated anatomically from the whole brain for each individual by means of the SUIT toolbox. As other non-cerebellar parts (e.g., the transverse sinus) were misclassified as parts of the cerebellum, an additional manual correction of the individual isolation map was necessary for most of the individuals.

For the functional analyses, the segmentations were normalized by using a non-linear deformation, whereas for the voxel-based morphometry (VBM) analyses, normalization of segmentations was carried out by using DARTEL (diffeomorphic anatomical registration using exponentiated lie algebra [22]). This step resulted in deformation maps, which then were used to reslice the functional images (voxel size 2 × 2 × 2 mm) and structural images (1 × 1 × 1 mm) to the SUIT atlas space. Finally, normalized images were smoothed with a Gaussian kernel of 6 mm.

In order to investigate connectivity patterns, the gPPI approach was used. It has been shown that this method is more sensitive and accurate than the “classic” PPI implemented in SPM [23]. A 4-mm sphere built around the activation peak (crus I; identified for the contrast pick–caress) was defined as seed region. The extracted time course for crus I was then used as a regressor in a GLM (general linear model) analysis.

In the second-level analysis step for the functional data, individual contrast images were entered into an analysis of variance (ANOVA) with the between-subject factor group (SPD patients/controls) and the within-subject factor condition (pick/caress/rest). Significant main effects and interactions were followed by post hoc *t* tests. For structural data and connectivity data, two-sample *t* tests were computed to investigate possible differences between SPD patients and controls. Furthermore, in a simple regression approach, MIDAS scores (automatic/focused picking) were used as criterion variables to capture the association with cerebellar activity (predictor variable) within the patient sample. Similarly, MIDAS scores were correlated with gray matter volumes (GMV) in cerebellar subregions for the patients.

For the gPPI analysis, group differences between connectivity maps of crus I and prefrontal regions of interest (ROIs: superior, middle, inferior frontal gyrus) were investigated (based on the findings by [12]). For all analyses, age was considered as a covariate of no interest. Furthermore, only for the SUIT analyses, a binarized explicit mask of the cerebellum and an additional application of an absolute threshold of 0.1 for the VBM analyses were used.

The current study used probabilistic ROI masks with a 50% threshold. Cerebellar masks were taken from the SUIT atlas. This resulted in ten cerebellar masks for each hemisphere and an additional mask for the vermis. All other ROI masks were taken from the Harvard-Oxford cortical and subcortical structural atlases. Results were considered significant if *p* < .05 corrected for family-wise error (FWE) on the voxel level (and cluster size > 10 voxels). All ROI results are small volume corrected.

## Results

### Brain Structure

#### VBM

**Fig. 1:**
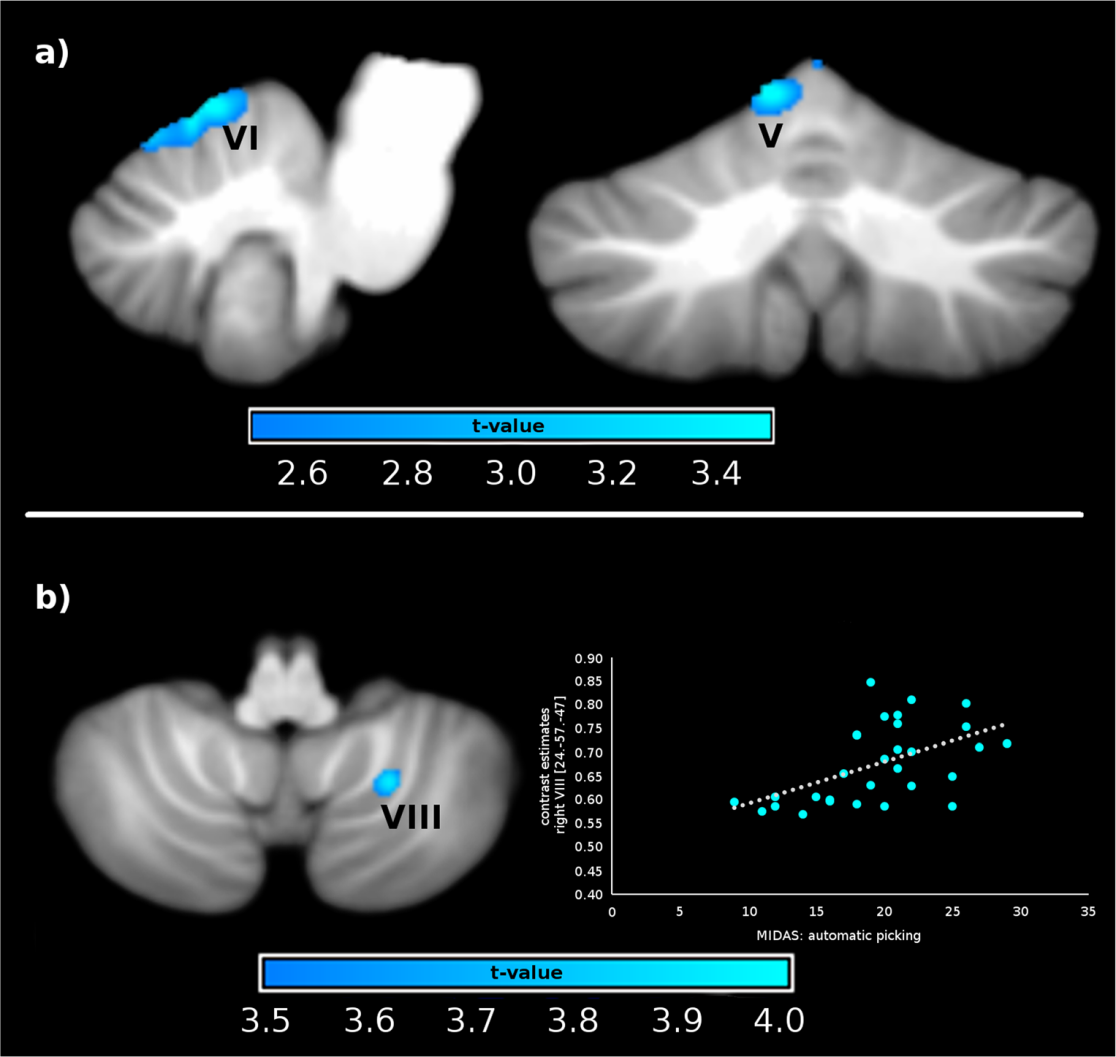
**a** Reduced gray matter volume (GMV) in lobules V and VI in SPD patients relative to controls. **b** Regression between patients’ automatic skin picking and GMV in lobule VIII. Automatic skin picking assessed with the Milwaukee Inventory for the Dimensions of Adult Skin Pick (MIDAS)

Relative to controls, SPD patients were characterized by reduced gray matter volumes in the left cerebellar lobules V (MNI coordinates *x*,*y*,*z*: − 5,− 67,− 11, *t* = 3.78, *p*(FWE) = .034, *d* = .99) and VI (MNI coordinates *x*,*y*,*z*: − 8,− 61,− 7, *t* = 3.69, *p*(FWE) = .026, *d* = .97; see Fig. 1).

Within the patient sample, the scores on the automatic picking scale (MIDAS) were positively associated with GMV in the right cerebellar lobule VIII (MNI coordinates *x*,*y*,*z*: 24,− 57,− 47, *t* = 4.20, *p*(FWE) = .016, *d* = 1.62, beta = .01; see Fig. 1).

### Brain Function

#### FMRI

**Fig. 2:**
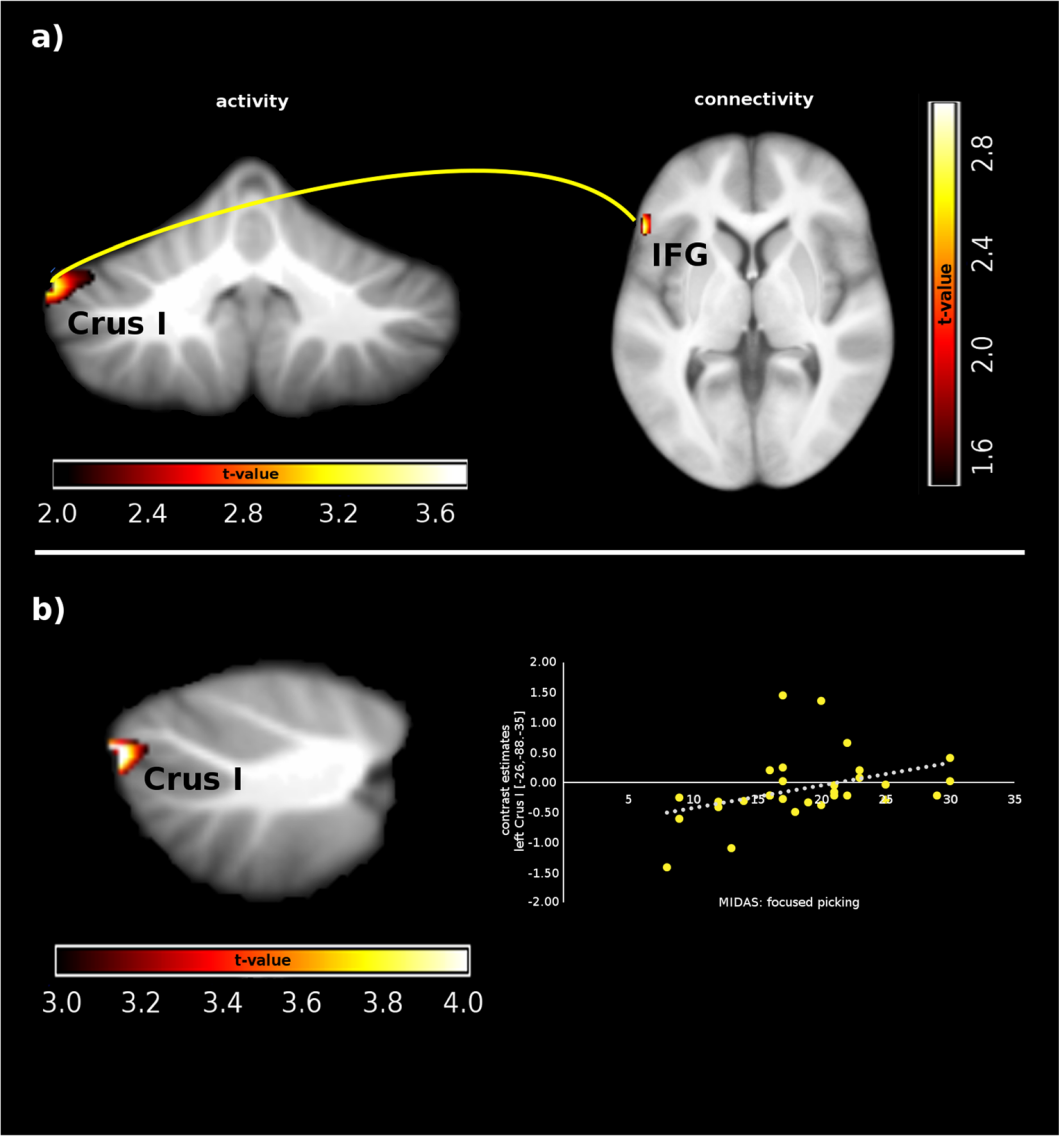
**a** Increased crus I activity/connectivity in SPD patients relative to controls during skin picking vs. caressing. **b** Regression between patients’ focused skin picking and crus I activity. IFG, inferior frontal gyrus; focused skin picking assessed with the Milwaukee Inventory for the Dimensions of Adult Skin Pick (MIDAS)

The ANOVA with the factors group (SPD/controls) and condition (pick/caress/rest) revealed significant main effects for group and condition as well as a significant interaction group × condition (all ps < .01). For the main effect, group post hoc *t* tests showed that SPD patients were characterized by less activity in the right VIIIa and VIIIb relative to controls. Post hoc *t* tests following the main effect condition revealed that caressing as well as picking relative to resting provoked enhanced activity in the left VIIIa. When caressing was contrasted with picking, increased activity in the left VIIIa, VIIIb, VIIb, and X was observed. Post hoc tests for the interaction effect showed that picking relative to caressing provoked increased activation of the left crus I in SPD patients relative to controls (Fig. 2). Detailed information can be found in Table 1.

**Table 1:** Results of the post hoc *t* tests for the ANOVA findings

	*H*	*x*	*y*	*z*	*t*	*p*(FWE)	*d*
Main effect group							
Controls–SPD patients							
VIIb	R	36	−60	−59	3.71	.0106	.98
*VIIIa*	*R*	*34*	*− 58*	*− 61*	*4.26*	*.0395*	*1.12*
Main effect condition							
Caress–rest							
*VIIIa*	*L*	*− 20*	*− 58*	*− 63*	*4.62*	*.0095*	*1.20*
Pick–rest							
*VIIIa*	*L*	*− 22*	*− 58*	*− 63*	*3.97*	*.0472*	*1.03*
Caress–pick							
VIIIa	L	− 26	− 46	− 47	3.49	.0227	.91
VIIIb	L	− 24	− 44	− 47	3.31	.0289	.86
VIIb	L	− 38	− 56	− 53	3.44	.0267	.90
X	L	− 24	− 40	− 45	2.61	.0340	.68
Interaction: group × condition							
(SPD: pick–caress) – (controls: pick–caress)							
Crus I	L	− 52	− 52	− 31	3.50	.0471	.92

Italicized data are the results of the whole brain analyses; normal, region of interest (ROI) findings; *H*, hemisphere; *x*,*y*,*z*, MNI coordinates; *p*(FWE): *p* value corrected for family-wise error; *d*, effect size Cohen’s *d*

Within the patient sample, the scores on the focused picking scale (MIDAS) were positively associated with activation in the left VI (MNI coordinates *x*,*y*,*z*: − 22,− 54,− 17, *t* = 3.52, *p*(FWE) = .031, *d* = 1.36, beta = .14) for the contrast pick–rest. Moreover, for the contrast pick–caress, MIDAS focused picking scores were positively associated with activation in the left crus I (MNI coordinates *x*,*y*,*z*: − 26,− 88,− 35, *t* = 4.84, *p*(FWE) = .047, *d* = 1.72, beta = .05).

#### gPPI

Based on the fMRI findings, crus I (left hemisphere) served as seed region. Picking relative to caressing was associated with increased connectivity of crus I with the left inferior frontal gyrus (MNI coordinates *x*,*y*,*z*: − 54,26,2, *t* = 2.88, *p* = .036, *d* = .76) in SPD patients relative to controls (Fig. 2). No other effects reach statistical significance.

## Discussion

The present study identified structural and functional cerebellar abnormalities in patients with skin-picking disorder (SPD). We used a cerebellum-optimized VBM protocol that allowed a highly sensitive investigation of cerebellar gray matter volume. This approach revealed reduced volumes in the left lobules V/VI for SPD patients.

Lobule V has predominantly sensorimotor functions. For example, Bushara [24] detected V activation during tactile stimulation of the hand. Lobule VI is engaged in tasks involving overt motor processes (e.g., finger tapping, articulation) but also coordinates higher level processes, such as spatial tasks, executive functions, and affective processing ([25–27]; for meta-analytic findings, see [14]). Narayanaswamy et al. [26] investigated medication-naïve patients with obsessive-compulsive disorder (OCD) and showed that the patients had a significantly smaller cerebellum compared to healthy controls, especially in lobule VI. According to DSM-5, SPD is classified as an OCD-related syndrome. Therefore, shared neurobiological features in OCD and SPD are to be expected. Critically, it has to be mentioned that reduced cerebellar volume has not been detected consistently in patients with obsessive-compulsive symptoms. A large meta-analysis of VBM studies reported increased cerebellar volume in OCD [28].

The degree of automatic picking (MIDAS) was positively associated with GMV of Lobule VIII in the patients. Previous studies showed that movement of the hand as well as tactile stimulation of the hand activated lobule VIII [14, 24, 29]. Therefore, it seems possible that a prolonged tactile arm/hand stimulation, which is typical for SPD, might lead to structural changes within the mentioned region.

In the functional MRI approach, the control group in contrast to SPD patients was characterized by a generally increased activation of cerebellar regions involved in affective (VIIb [30, 31]) and motor-related (VIII [14]) tasks. The latter subregion also showed enhanced activity in the total sample when comparing caressing and picking with the rest condition. This observed cerebellar activity might reflect motor-related processes required for carrying out the tasks. The contrast caress–pick revealed enhanced activity in the vestibular region X together with activity in regions implicated in motor and affective processing (VII, VIII). This indicates that caressing relative to picking obviously was more pleasant and required more subtle and complex motion than picking.

Interestingly, although SPD patients were characterized by diminished overall cerebellar activation, they displayed increased activation of the left cerebellar crus I during skin picking vs. caressing when compared with controls. According to a meta-analysis with data for healthy participants [14], crus I and II are involved in cognitive functions (e.g., verbal working memory, executive functions). In addition, it was demonstrated that activation specific to emotional processing can be found in lobules VI and IV/V and bilateral crus 1. The present study identified a specific role of crus I for skin picking because patients’ scores on the focused picking scale (MIDAS) were positively associated with activation in this region.

Another meta-analysis on the role of the cerebellum in social cognition, identified crus I activation during mentalizing about the self, close others, and distant others as well as during abstract mentalizing (e.g., projecting oneself into the future and recalling the autobiographical past [15]). The authors of this meta-analysis argued that the cerebellum does not play a specific role in social cognition, but provides executive and semantic support for this function. Consequently, the cerebellum has a modulatory role; it updates information and sends adaptive feedback to the cerebral cortex, including prefrontal regions.

In line with this assumption, a group difference in cerebellar connectivity with a prefrontal region was observed in the present analysis. During skin picking, SPD patients showed enhanced coupling of the left crus I with the left inferior frontal gyrus. This region belongs to the ventrolateral prefrontal cortex (VLPFC), which is central for cognitive and affective control. For example, emotion regulation recruits the VLPFC besides other prefrontal regions [31]. The authors studied two strategies for affect control: reappraisal (a cognitive-linguistic strategy that alters emotional responses by reformulating the meaning of a situation) and expressive suppression (a strategy directed toward inhibiting affective behaviors such as facial expressions or verbal utterances). The participants used these two emotion regulation strategies during the presentation of affective film clips. Relative to passive viewing (and reappraisal), attempts of suppression were associated with VLPFC activation.

It has been argued that emotional dysregulation is one core pathological mechanism in SPD [32, 33]. Skin-picking has been conceptualized as a maladaptive mechanism of affect control [1]. Prior to the skin manipulation, the patients perceive tension or negative emotional states. The intensity of these aversive states is reduced—at least temporarily—via the picking. The increased cerebellum-VLPFC coupling possibly reflects increasing efforts to exert more cognitive control to counter neuronal input from the cerebellum.

Finally, the following shortcomings of the present study need to be considered. The patient sample was not representative but consisted of diagnostic-/treatment-seeking persons. Moreover, the patient group was relatively small and only included 11 men (but 19 women). Therefore, no gender comparisons could be conducted. The total sample was characterized by a large variance in age, which was used as a covariate in the analyses. Future studies should specifically investigate effects of age, focus on possible gender differences, and should include additional clinical comparison groups (e.g., patients displaying other body-focused repetitive behaviors or OCD-related symptoms). Furthermore, different picking groups could be compared (e.g., high vs. low automatic/focused picking) in order to identify underlying neuronal correlates. Finally, longitudinal approaches are needed to answer questions about changes regarding cerebellar structure/activity over time.

In summary, this study provides first evidence of structural as well as functional changes in specific subregions of the cerebellum related to pathological skin picking.
